# An Engineered Mouse to Identify Proliferating Cells and Their Derivatives

**DOI:** 10.3389/fcell.2020.00388

**Published:** 2020-05-25

**Authors:** Jihyun Jang, Kurt A. Engleka, Feiyan Liu, Li Li, Guang Song, Jonathan A. Epstein, Deqiang Li

**Affiliations:** ^1^Department of Surgery, Center for Vascular and Inflammatory Diseases, University of Maryland School of Medicine, Baltimore, MD, United States; ^2^Department of Cell and Developmental Biology, Perelman School of Medicine, University of Pennsylvania, Philadelphia, PA, United States; ^3^Penn Cardiovascular Institute, Perelman School of Medicine, University of Pennsylvania, Philadelphia, PA, United States

**Keywords:** cell proliferation, aurora kinase B, mouse model, lineage tracing, regeneration, development

## Abstract

**Background:**

Cell proliferation is a fundamental event during development, disease, and regeneration. Effectively tracking and quantifying proliferating cells and their derivatives is critical for addressing many research questions. Cell cycle expression such as for Ki67, proliferating cell nuclear antigen (PCNA), or aurora kinase B (Aurkb), or measurement of 5-bromo-2′-deoxyuridine (BrdU) or ^3^H-thymidine incorporation have been widely used to assess and quantify cell proliferation. These are powerful tools for detecting actively proliferating cells, but they do not identify cell populations derived from proliferating progenitors over time.

**Aims:**

We developed a new mouse tool for lineage tracing of proliferating cells by targeting the *Aurkb* allele.

**Results:**

In quiescent cells or cells arrested at G1/S, little or no *Aurkb* mRNA is detectable. In cycling cells, *Aurkb* transcripts are detectable at G2 and become undetectable by telophase. These findings suggest that *Aurkb* transcription is restricted to proliferating cells and is tightly coupled to cell proliferation. Accordingly, we generated an *Aurkb*^*ER Cre/+*^ mouse by targeting a tamoxifen inducible Cre cassette into the start codon of *Aurkb*. We find that the *Aurkb*^*ER Cre/+*^ mouse faithfully labels proliferating cells in developing embryos and regenerative adult tissues such as intestine but does not label quiescent cells such as post-mitotic neurons.

**Conclusion:**

The *Aurkb*^*ER Cre/+*^ mouse faithfully labels proliferating cells and their derivatives in developing embryos and regenerative adult tissues. This new mouse tool provides a novel genetic tracing capability for studying tissue proliferation and regeneration.

## Introduction

Cell proliferation is a fundamental biological event in all multicellular organisms (Nasmyth, 2001). Identification or quantification of proliferating cells is essential to understanding organogenesis, morphogenesis, tumorigenesis, and regeneration. Replicating cells can be identified based on expression of cell-cycle markers, such as Ki67, proliferating cell nuclear antigen (PCNA), aurora kinase B (Aurkb), or the incorporation of thymidine analogs, such as ^3^H-thymidine, 5-bromo-2′-deoxyuridine (BrdU), or 5-ethynyl-2′-deoxyuridine (EdU) (Mitchison and Salmon, 2001). Incorporation of 5-iodo-2′-deoxyuridine (IdU) has been used to analyze proliferation in human tissue (Pan et al., 2013). These assays are suitable for detecting actively proliferating or label-retaining cells. However, retrospective lineage tracing is often desired when proliferating cells must be tracked for their growth pattern or quantity under certain biological conditions such as tissue morphogenesis or regeneration.

Genetic engineering in the mouse allows lineage tracing mouse models to track the derivatives of proliferating cells. One model is the mosaic analysis with double markers (MADM) mouse model, which labels dividing cells through interchromosomal recombination (Zong et al., 2005), although its application is limited due to low labeling efficiency. More recently, a Ki67^IRESCreER/+^ mouse was generated and used to track proliferating cells in brain or heart (Basak et al., 2018; Kretzschmar et al., 2018). However, Ki67 is expressed throughout the cell cycle including G1, and some non-proliferative cells such as adult cardiomyocytes can poise at G1 for an extended period of time without cell division (Alvarez et al., 2019).

Aurkb, a key component of the chromosomal passenger complex, localizes to the centromeres to ensure precise chromosome segregation during mitosis and to the midbody to assist cytoplasmic separation during cytokinesis (van der Waal et al., 2012). Knockdown or inhibition of Aurkb *in vitro* inhibits cell proliferation (Yu et al., 2015; Helfrich et al., 2016), while knockout of *Aurkb* in mice results in mitotic defects in the inner cell mass (Fernandez-Miranda et al., 2011). Increased expression of Aurkb is associated with tumorigenesis and inhibition of Aurkb may be an effective cancer therapeutic target (Tang et al., 2017; Tischer and Gergely, 2019). Aurkb has been widely used to identify mitotic cells using immunofluorescence or immunohistochemical methods with anti-Aurkb antibodies (Vader and Lens, 2008; Liu and Lampson, 2009; van der Waal et al., 2012; Tian et al., 2015; Nakada et al., 2017; Yu et al., 2019).

In order to track cell proliferation retrospectively, we have generated *Aurkb*^*ER Cre/+*^ mice by targeting a tamoxifen inducible Cre cassette into the start codon of *Aurkb*. By characterizing the *Aurkb*^*ER Cre*^ allele *in vitro* and *in vivo*, we show that *Aurkb*^*ER Cre/+*^ mice faithfully label proliferating cells and their derivatives during development and regeneration.

## Materials and Methods

### Mice

*Aurkb*^*ER Cre/+*^ mice were generated by homologous recombination in embryonic stem cells targeting a Cre-Ert2-V2A-tdTomato-Frt-PGK-neo-Frt cassette into the start codon of the *Aurkb* locus. Thus, the insertion of this cassette will lead to the ablation of endogenous *Aurkb* expression in the target allele. The PGK-Neo cassette was removed by breeding the initial progeny to mice expressing ubiquitous FlpE recombinase (Rodriguez et al., 2000). Southern blot confirmed the expected homologous recombination and germ line transmission of the targeted allele. The *Aurkb*^*ER Cre*^ allele is detected by PCR using the following primers: Forward: 5′-GTGGGCTCTATGGCTTCTGA-3′, Reverse (common): 5′-CAAATTCTTGAGGCCCACAC-3′; product size: 501 bp. The wild-type allele is detected by using the following primers: Forward: 5′-ATGGACCTAGAGCGGGAGAT-3′ and Reverse (common); product size: 264 bp. The V2A-tdTomato included in the targeting construct potentially provides a means to fluorescently label *Aurkb*-expressing cells without disrupting Cre-Ert2 function. However, although we were able to detect tdTomato protein expression by immunofluorescence using antibodies on fixed intestinal crypts (Supplementary Figure 1), the spontaneous tdTomato fluorescence was below levels of detection. B6.129 × 1-*Gt (ROSA) 26Sor^TM 1(EYFP)Cos/+^* (abbreviated as *R26R*^*eYFP*^) mice were purchased from The Jackson Laboratory (stock number: 006148). All mice were maintained on a mixed genetic background. All animal protocols were approved by the University of Pennsylvania Institutional Animal Care and Use Committee (IACUC #: 803396) and the University of Maryland Baltimore Institutional Animal Care and Use Committee (IACUC #: 0118005).

### Administration of Tamoxifen and 5-Bromo-2′-Deoxyuridine (BrdU) *in vivo*

Tamoxifen (Sigma-Aldrich, St. Louis, MO, United States) (10 mg/ml) was dissolved in corn oil. Tamoxifen [2 or 100 or 150 mg/kg body weight (BW)] was given to *Aurkb*^*ER Cre/+*^; *R26R*^*eYFP/+*^ mice by either intraperitoneal injection or gavage. BrdU (Sigma-Aldrich, St. Louis, MO, United States) (10 mg/ml) was dissolved in phosphate-buffered saline (PBS) and intraperitoneally delivered to *Aurkb*^*ER Cre/+*^; *R26R*^*eYFP/+*^ mice (100 mg/kg BW).

### Histology, Immunofluorescence and RNAscope

All specimens for paraffin sections were fixed in 4% (w/v) paraformaldehyde (PFA) overnight, dehydrated through an ethanol series, paraffin embedded, and sectioned (6–7 μm). Primary antibodies (Supplementary Table 1) were incubated at 4°C overnight and secondary antibodies (Alexa 488, 555, or 647, Life Technologies, Grand Island, NY, United States) were incubated at room temperature for 1 h. The *Aurkb* RNAscope probe (173–1483 bp of the *Mus musculus Aurkb* mRNA sequence) was designed and provided by Advanced Cell Diagnostics (Hayward, CA, United States). RNAscope *in situ* hybridizations (Ikpa et al., 2016) were performed according to the protocol provided by manufacturer.

### Image Analysis and Quantification

ImageJ software was used for quantification of GFP+ and/or BrdU+ cells on histology slides. Samples from 3–6 mice each were counted at any given time point or condition. The reported values represent the mean score.

### Live Cell Imaging

Time-lapse phase-contrast and GFP immunofluorescence images of mouse embryo fibroblasts (MEFs) were taken for 22 h after 4-OH tamoxifen induction (final concentration: 1 μg/ml) by using the IncuCyte live-cell culture system (Essen Bioscience). The images were then analyzed and converted to movie format by using IncuCyte software.

### Fluorescence-Activated Cell Sorting (FACS) Analyses

MEFs were isolated and cultured as previously described (Li et al., 2011). MEFs were treated with either control vehicles or designated cell cycle inhibitors, then digested and collected as single cell suspensions. The cell suspension was washed with PBS and then fixed with intracellular fixation buffer (eBiosciences). For intracellular FACS analyses, cells were permeabilized with permeabilization buffer (eBiosciences) and then incubated with GFP antibodies (see Supplementary Table 1) for 2 h at room temperature, followed by incubation with secondary antibodies (Alexa fluor, Life Technologies) for 1 h at room temperature. Samples were run and analyzed using a BD FACS Canto II instrument and software (BD Biosciences).

### Quantitative Real-Time PCR (qRT-PCR)

Heart, brain, and embryonic tissues were microdissected in cold PBS and snap frozen in liquid nitrogen. TRIzol reagent (Life Technologies, Grand Island, NY, United States) was used to extract total RNA and complementary DNA (cDNA) was generated with the Superscript III kit (Life Technologies, Grand Island, NY, United States). SYBR Green quantitative RT-PCR was performed using the StepOne Plus Real-Time PCR System (Applied Biosystems, Foster City, CA, United States). Primers for *Aurkb*: P1F (forward): 5′-TCGCTGTTGTTTCCCTCTCT-3′, P1R (reverse): 5′-TTCAGGCCAGACTGAGACG-3′; P2F (forward): TCGCTGTTGTTTCCCTCTCT, P2R (reverse): TTCAGGCCAGACTGAGACG. Primers for *Gapdh*: Forward: 5′-TCTTGCTCAGTGTCCTTGCTGG-3′, Reverse: 5′-TCCTGGTATGACAATGAATAC GGC-3′.

### Western Blotting

E12.5 embryos were minced in cold lysis buffer (50 mM Tris–HCl (pH 7.4), 150 mM NaCl, 1 mM EDTA-Na_2_, 1 mM EGTA, 1% Triton X-100, 0.5% Sodium Deoxycholate and 0.1% SDS with Protease inhibitor cocktail (Roche); 1 mM phenylmethylsulfonyl fluoride was added before use). Protein samples were resolved on 4–12% SDS-PAGE acrylamide gel before transferring to PVDF membranes. We used primary antibodies to Aurkb (1:1000), Cre (1:1000) and GAPDH (1:5000). Primary antibodies were visualized by chemiluminescence using HRP-conjugated secondary antibodies.

### Statistical Analysis

Data are presented as mean ± SEM. Statistical significance between two groups was determined using two-tailed Student’s *t*-test or chi square test. If significance is to be tested between multiple groups, an analysis of variance is performed, followed by Bonferroni *post hoc* test. *P* < 0.05 was considered significant.

## Results

### *Aurkb* Is Expressed in Proliferating but Not in Quiescent Cells

**FIGURE 1:**
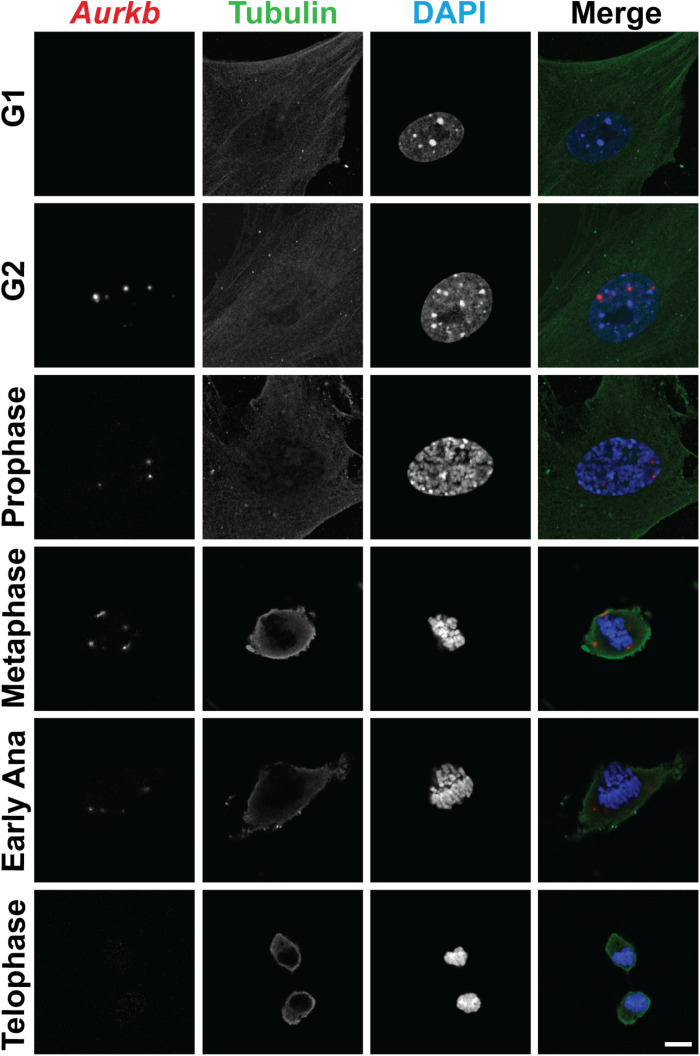
The expression of *Aurkb* transcripts during the cell cycle. Double staining of RNAscope and immunofluorescence in MEFs. Bar, 10 μm.

**FIGURE 2:**
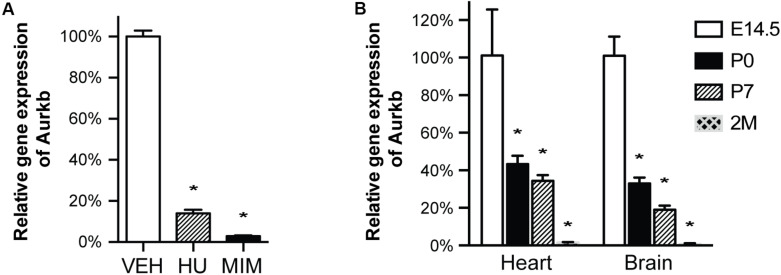
*Aurkb* transcription is coupled with cell proliferation. **(A)** MEFs were cultured for 48 h with normal media (control), or treated with hydroxyurea (2 mM), or mimosine (1 mM). The transcriptional levels of *Aurkb* were quantified by qRT-PCR. *Gapdh* was used as a cDNA loading control. Three independent biological samples were used in each condition. *P < 0.05 when compared to the control condition analyzed by ANOVA followed by Bonferroni *post hoc* test; VEH, vehicle control; HU, hydroxyurea; MIM, mimosine; **(B)** The relative transcriptional levels of *Aurkb* in heart and brain at E14.5, postnatal day 0 (P0), postnatal day 7 (P7), and 2 months (2M). *Gapdh* was used as a cDNA loading control. *n* = 3 in each group. *P < 0.05 when compared to E14.5 analyzed by ANOVA followed by Bonferroni *post hoc* test.

**FIGURE 3:**
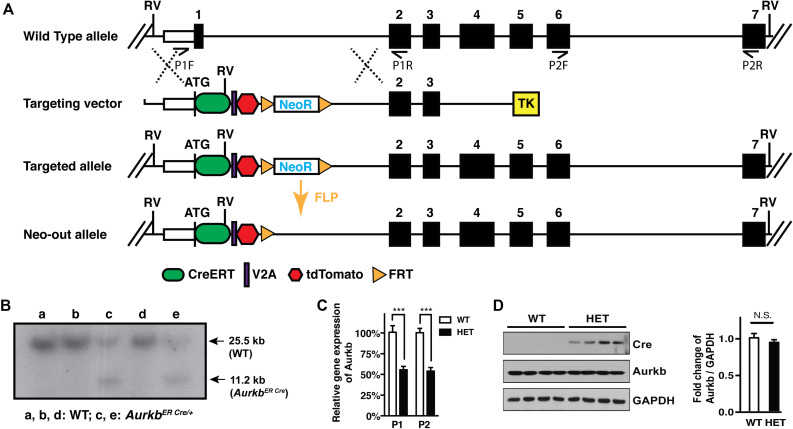
*Aurkb* gene targeting. **(A)** Schematic of the generation of the *Aurkb*^*ER Cre*^ allele; **(B)** Southern blot of DNA extracted from *Aurkb*^*ER Cre/+*^ MEFs. DNAs were digested by *Eco*RV (RV). The ^32^P-radiolabeled 5′ DNA probe used (∼500 bp) is located upstream of the 5′ arm but downstream to the 5′ RV enzyme site. P1F, P1R, P2F, and P2R are primer sets for detecting *Aurkb* transcripts; **(C)** *Aurkb* mRNA expression in wildtype (WT) and heterozygous (HET) E12.5 embryos. *n* = 4 in each group. ***P < 0.001 by a Student’s *t*-test; **(D)** *Aurkb* protein expression in WT and HET E12.5 embryos. GAPDH was used as a protein loading control. Densitometric quantification of Aurkb was shown on the right. N.S., not significant.

In cultured MEFs, Aurkb protein is undetectable at G1 phase, but expression becomes prominent at G2 and it is localized to the nucleus. *Aurkb* reaches and maintains a strong expression level throughout M phase. *Aurkb* re-localizes to the midbody at telophase (Supplementary Figure 2). These findings are consistent with previous observations (Crosio et al., 2002; Li et al., 2015). *Aurkb* mRNA is not detectable at G1 but is detectable by G2. Message is present through M phase but becomes undetectable at telophase (Figure 1). These results suggest that *Aurkb* transcript expression is correlated with the phase of the cell cycle and is largely restricted to mitotic cells. To further test this association, we forced MEFs to arrest at G1/S phase by exposure to hydroxyurea or mimosine (Park et al., 2012) and then assessed the presence of *Aurkb* transcripts. *Aurkb* transcriptional levels were significantly decreased in hydroxyurea- and mimosine-treated groups as compared to the control group (Figure 2A). Both brain and heart experience a proliferation transition from being highly proliferative at embryonic stage to being mostly proliferatively inert in adulthood (Brooks et al., 1998). *Aurkb* transcription significantly declines from being high at embryonic day (E) 14.5 to being almost undetectable in adult heart and brain (Figure 2B). Altogether, these data suggest that *Aurkb* transcription is coupled with cell proliferation *in vitro* and *in vivo*. Accordingly, we generated *Aurkb*^*ER Cre/+*^ mice by targeting a Cre-Ert2 cassette into the start codon of the *Aurkb* locus (Figure 3A). As expected, *Aurkb* mRNA was reduced to about 50% in *Aurkb*^*ER Cre/+*^ heterozygous mice as compared to their wildtype littermate controls (Figure 3C). In contrast, *Aurkb* protein expression was similar between *Aurkb*^*ER Cre/+*^ heterozygous mice and wildtype controls (Figure 3D). *Aurkb*^*ER Cre/+*^ heterozygous mice are phenotypically normal and fertile. The total knockout of *Aurkb* resulted in early post-implantation lethality by E9.5 (Fernandez-Miranda et al., 2011). Consistently, we did not recover any *Aurkb*^*ER Cre/ER Cre*^ embryos at E10.5 (0/21).

### *Aurkb*^*ER Cre*^ Labels Proliferating Cells *in vitro*

**FIGURE 4:**
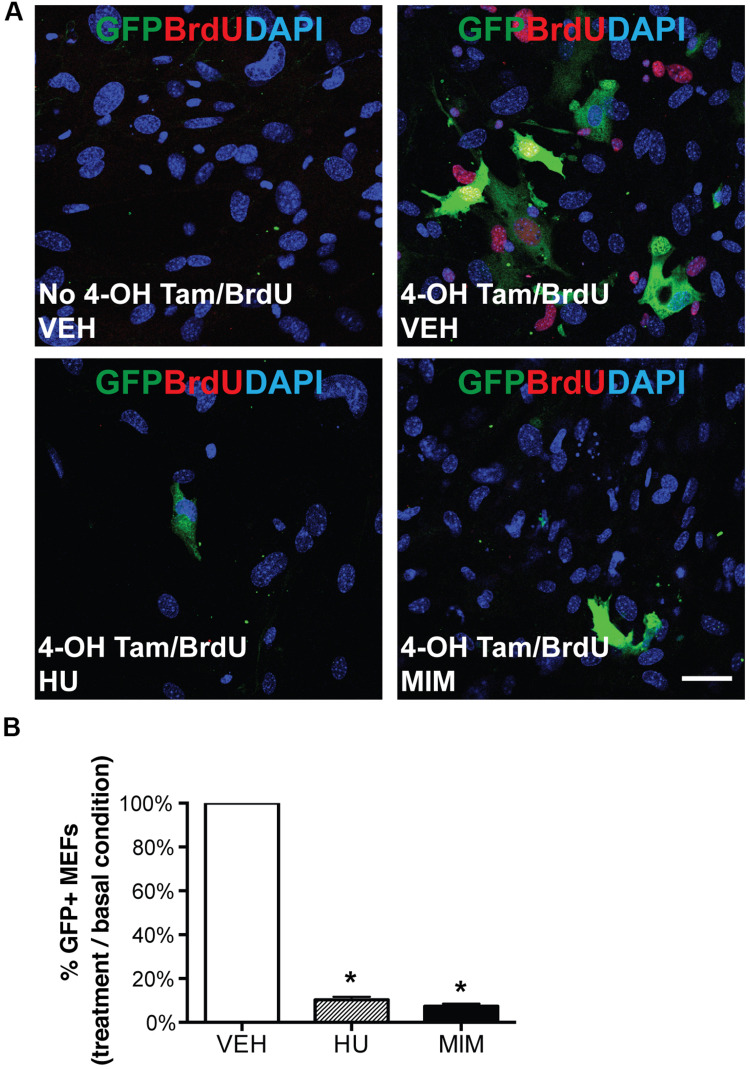
*Aurkb*^*ER Cre/+*^ labeling of MEFs. *Aurkb^*ER*^^*Cre/+*^; R26R*^*eYFP/+*^ MEFs were cultured in 10% FBS standard medium. MEFs were treated with cell cycle blockers for a total of 48 h. 24 h after the treatment, BrdU (10 μM) and 4-OH tamoxifen (1 μM) were added in the medium and maintained for 24 h before cell harvest. YFP immunosignal was detected by GFP antibody. **(A)** Representative micrographs of BrdU and GFP immunofluorescence staining of *Aurkb^*ER*^^*Cre/+*^; R26R*^*eYFP/+*^ MEFs under various culture conditions. Scale bar, 50 μm; **(B)** The *Aurkb^*ER*^^*Cre/+*^* labeling percentage was quantified by flow cytometric analysis. The percentage was calculated as the number of GFP + cells under each treatment condition as divided by the number of GFP + cells in the control condition. VEH, vehicle control; HU, hydroxyurea (2 mM); MIM, mimosine (1 mM). *n* = 3 for each condition, *P < 0.05 when compared to the control group by ANOVA followed by Bonferroni *post hoc* test.

To characterize the labeling of *Aurkb*^*ER Cre*^ *in vitro* and test whether it is associated with cell proliferation, we generated *Aurkb*^*ER Cre/+*^; *R26R*^*eYFP/+*^ MEFs and tracked *Aurkb*^*ER Cre*^ labeling by following the YFP reporter activities after 4-OH tamoxifen induction. YFP signal was detectable in proliferating MEFs about 16 h after 4-OH tamoxifen induction, became strong immediately prior to cell division, and maintained expression in daughter cells (Supplementary Video 1). In contrast, there was no YFP signal in non-dividing *Aurkb*^*ER Cre/+*^; *R26R*^*eYFP/+*^ MEFs. Note that YFP signal is well recognized by GFP antibodies. Hereafter, we use GFP antibodies to measure YFP expression when referring to *Aurkb*^*ER Cre/+*^ fate-mapped cells. There was negligible *R26R*^*eYFP/+*^ reporter activity in *Aurkb*^*ER Cre/+*^; *R26R*^*eYFP/+*^ MEFs without 4-OH tamoxifen induction (Figure 4A), indicating that there is little to no leakiness of the *Aurkb*^*ER Cre/+*^ allele. According to the expression profile of *Aurkb*, we expected to see *Aurkb*^*ER Cre/+*^ labeling cells as they enter G2 phase. To further analyze the association between *Aurkb*^*ER Cre/+*^ labeling and cell proliferation, we arrested MEFs at G1/S phase by either hydroxyurea or mimosine treatment, as evidenced by the absence of BrdU incorporation (Figure 4A). *R26R*^*eYFP/+*^ reporter activities were significantly lower for cell cycle inhibitor-treated *Aurkb*^*ER Cre/+*^; *R26R*^*eYFP/+*^ MEFs compared to those under normal culture conditions (Figures 4A,B). This lineage tracing result mirrors the *Aurkb* transcription profile when wild-type MEFs are arrested at G1/S phase (Figure 2A). These results suggest that *Aurkb*^*ER Cre*^ labels proliferating but not non-dividing cells *in vitro*.

### *Aurkb*^*ER Cre*^ Labels Proliferating Cells During Embryonic Development

**FIGURE 5:**
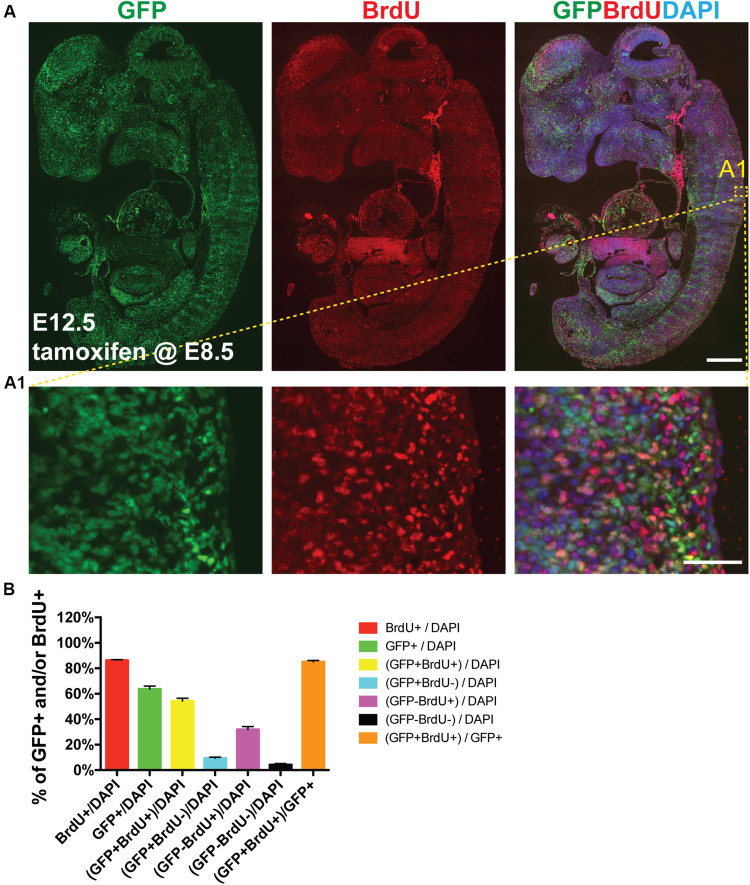
*Aurkb*^*ER Cre/+*^ labels proliferating cells in developing embryos. Tamoxifen (150 mg/kg BW) was given to pregnant mice at E8.5 by gavage. BrdU (100 mg/kg BW) was intraperitoneally given to pregnant mice for four consecutive days (E8.5, E9.5, E10.5, and E11.5, one injection/day). **(A** and **A1)** Representative immunofluorescence micrographs of an E12.5 *Aurkb^*ER Cre/+*^; R26R^*eYFP/+*^* embryo (sagittal section). Scale bars, A, 500 μm; A1, 50 μm; **(B)** Labeling quantification of *Aurkb*^*ER Cre/+*^ and/or BrdU (*n* = 4).

Next we sought to determine whether *Aurkb*^*ER Cre*^ labels highly proliferative cells during embryonic development *in vivo*. We confirmed that *Aurkb* heterozygosity did not grossly affect embryonic morphogenesis or cellular growth (Supplementary Figure 3), validating the use of *Aurkb*^*ER Cre/+*^ as a lineage tracing tool during embryonic development. When E8.5 *Aurkb*^*ER Cre/+*^; *R26*^*eYFP/+*^ embryos were induced with tamoxifen, extensive labeling of the embryo was observed. Importantly, there was no leakiness of *Aurkb*^*ER Cre*^ labeling when corn oil but not tamoxifen was administered (Supplementary Figure 4). When we labeled developing embryos with both *Aurkb*^*ER Cre*^ and BrdU, we found that about 85% of embryonic cells are labeled by both systems (Figure 5). Further, we performed double immunofluorescence staining of GFP and PCNA on these embryos. We found that nearly 93% of embryonic cells are double positive (Supplementary Figure 5). Altogether, these data indicate that *Aurkb*^*ER Cre*^ is a sensitive and reliable system for lineage tracking of proliferating embryonic cells.

### Tamoxifen Activation of *Aurkb*^*ER Cre*^ Labels Proliferating Adult Stem/Progenitor Cells but Not Post-mitotic Cells *in vivo*

**FIGURE 6:**
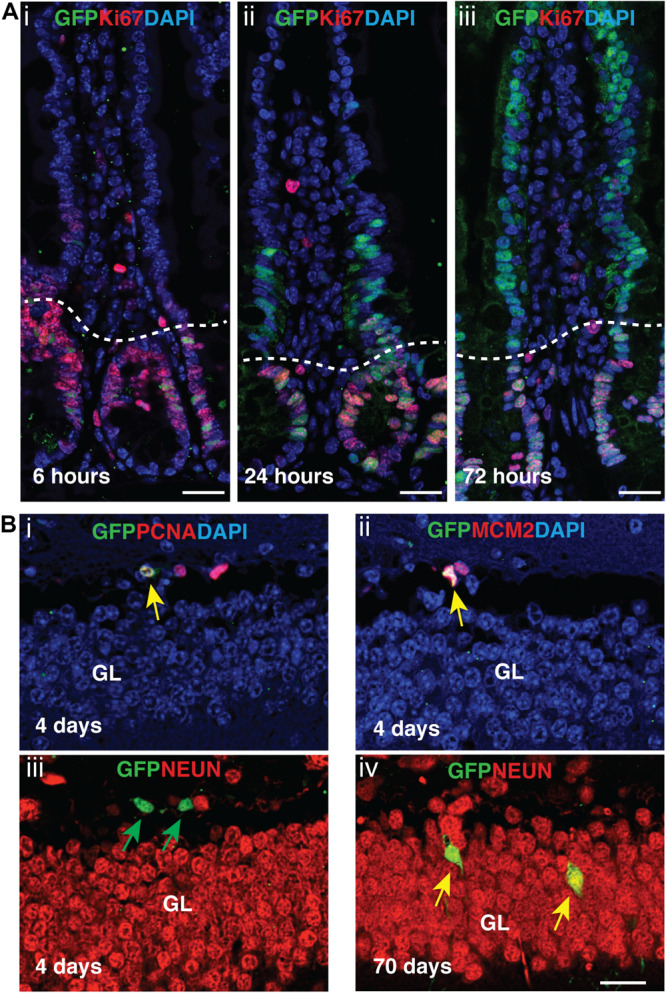
Tamoxifen activation of *Aurkb*^*ER Cre/+*^ labels proliferating stem cells/progenitor cells in adult intestines and brain. **(A)** *Aurkb*^*ER Cre/+*^ labeling in adult intestines. A single dose of tamoxifen (100 mg/kg BW, i–iii) was intraperitoneally given to *Aurkb^*ER Cre/+*^; R26R^*eYFP/+*^* mice at the age of 2 months. Dotted lines delineate the crypts below from their associated villus above. Bars, 25 μm; **(B)** *Aurkb*^*ER Cre/+*^ labeling in adult brain. Tamoxifen (100 mg/kg BW) was intraperitoneally given daily to *Aurkb^*ER Cre/+*^; R26R^*eYFP/+*^* mice for four consecutive days at the age of 2 months. Green arrows point to GFP+ only cells; yellow arrows point to GFP+; PCNA+ (i), GFP+; MCM2+ (ii) or GFP+; NeuN+ (iv) cells. GL, granular layer. Bars, 20 μm.

Next, we assessed *Aurkb*^*ER Cre*^ labeling in adult regenerative tissues. Two-month-old *Aurkb^*ER Cre/+*^; R26^*eYFP/+*^* mice were given a single dose of tamoxifen (100 mg/kg BW) and we followed the labeling pattern of the YFP reporter over time in the intestine. The labeling displayed a dynamic expansion from the initial crypt (6 h after tamoxifen administration) to the entire crypt-villus structure over a course of 3 days (Figure 6A), greatly resembling the lineage tracking pattern of intestinal stem cells (ISCs) and progenitors, which are known to be highly proliferative (Barker, 2014). In the crypt zone where Ki67-positive ISCs and progenitors reside, *Aurkb*^*ER Cre*^ labeling colocalizes with Ki67. In the villi where derivative, Ki67-negative intestinal epithelial cells reside, *Aurkb*^*ER Cre/+*^ labeling colocalizes with derivatives of the crypt ISCs that appear over time (Figure 6A). Further, administration of low doses of tamoxifen (2 mg/kg BW) in adult *Aurkb*^*ER  Cre/+*^; *R26R^*eYFP*/+^* mice revealed that single earlier *Aurkb*-labeled intestinal progenitor cells (PCNA+) expanded to form clusters of enterocytes after 7 days (Supplementary Figure 6). Altogether, these data indicate that *Aurkb*^*ER Cre/+*^ labels proliferating progenitor cells in the intestine. In brain, neuronal nuclei (NeuN)-positive post-mitotic neurons were not promptly labeled after tamoxifen exposure but eventually became labeled (70 days after the initial tamoxifen induction). This occurred presumably through transit amplifying mini-chromosome maintenance proteins (MCM2)-positive progenitors (Figure 6B). This is consistent with NeuN-positive neurons as terminally quiescent cells, renewing through neural stem cells and progenitors with prolonged kinetics.

## Discussion

Assessment of cell proliferation *in vivo* generally relies on the use of BrdU incorporation or “snapshots” of cell cycle marker expression such as PCNA or Ki67. Both approaches are important complementary methods for detecting actively proliferating cells. However, increased focus on adult tissue regeneration calls for new tools to enable the detection of tissues and cell lineages derived from proliferating adult progenitor cells under various conditions. The MADM mouse can be used for tracking populations derived from proliferative progenitors. However, its application is limited due to its low detection sensitivity (Zong, 2014). A *Ki67*^*IRESCreER*^ mouse tracks proliferating cells based on Ki67 expression (Basak et al., 2018). However, Ki67 is transcribed broadly throughout the cell cycle, a feature that appears a critical factor in its reliability as a proliferation marker (Miller et al., 2018). In this report, we describe a new genetic mouse tool, *Aurkb*^*ER Cre/+*^, which can label cells based on *Aurkb* transcription. We found that *Aurkb*^*ER Cre*^ labels proliferating cells *in vitro* and *in vivo*. In developing embryos, *Aurkb*^*ER Cre*^ labeling overlaps well with cell proliferation markers such as BrdU and PCNA but it does not label quiescent cells. We notice that the overlap between *Aurkb*^*ER Cre*^ labeling and BrdU or PCNA seems quite high during development, even though *Aurkb*^*ER Cre*^ labeling was induced by a short pulse of tamoxifen. This is primarily due to the high proliferative characteristics of developing embryos: both earlier proliferating embryonic cells and their later derivatives are all mitotic.

Since *Aurkb*^*ER Cre*^ labeling is based on *Aurkb* transcription, which turns on at G2 phase, *Aurkb^*ER* Cre^* labeling should not be interpreted as an absolute cell division or cytokinetic marker. For instance, certain cells such as bi- or multinucleated adult cardiomyocytes and hepatocytes can undergo karyokinesis without cytokinesis under physiological or pathological conditions (Gentric et al., 2012; Derks and Bergmann, 2020). These cells may be labeled by *Aurkb*^*ER Cre*^ in the absence of cell division. In these specific instances, *Aurkb* protein expression and other cytokinetic events should be analyzed instead.

Since *Aurkb*^*ER Cre/+*^ is built by a knock-in strategy, the *Aurkb* transcript is disrupted in the targeted allele. In contrast, *Aurkb* protein expression level in *Aurkb*^*ER Cre/+*^ heterozygous mice was quite similar to their wildtype controls (Figure 3D). This is consistent with other reports that heterozygous knockout mice can express similar or more than half of the proteins relative to wildtype control mice in the target genes (Gineste et al., 2013; Zhou et al., 2015; Arkhipov et al., 2019). It is reported that an *Aurkb* knockout mouse in which exons 2–6 were excised is embryonic lethal, older *Aurkb* heterozygous mice approximately 12–24 months of age show decreased survival due to susceptibility for tumorigenesis, and a fraction of *Aurkb* heterozygous males suffer from oligospermia by 12 months of age (Fernandez-Miranda et al., 2011). In our current study, we used much younger mice less than 3-months of age. We did not observe reproductive defects or spontaneous cancer development in young *Aurkb*^*ER Cre/+*^ mice. Nonetheless, it is possible that *Aurkb*^*ER Cre/+*^ mice may develop aforementioned pathologies over time, due to genome or chromosomal instability, even though we did not observe such problems in our colony. On the other hand, by intercrossing *Aurkb*^*ER Cre/+*^ heterozygous mice, the progeny with genotype of *Aurkb*^*ER Cre/ER Cre*^ can be used to study the phenotype of *Aurkb* depletion or requirement of *Aurkb* expression in cell division and cytokinesis.

The *Aurkb*^*ER Cre/+*^ mouse is an important new tool for lineage tracing proliferating cells during embryonic development and adult tissue regeneration. For instance, when crossed to multi-color reporters such as the confetti mouse strain (Snippert et al., 2010), the *Aurkb*^*ER Cre/+*^ mouse can be used to study the clonogenicity of neurons or other cell types in the developing embryos or tissue regeneration (e.g., whether cells are derived from multiple progenitor cells or from rare dominant clones). Similarly, the *Aurkb*^*ER Cre/+*^ mouse can be used to track tissue regeneration such as occurring from those rare stem cells whose derivatives forming over long periods of time will be more readily detected with this tool. The *Aurkb*^*ER Cre/+*^ mouse allows the ability to track how tissues with low rates of proliferation such as brain, lung and heart are regenerated over time in various physiological or pathological conditions. We anticipate the coupling of *Aurkb* knockout with inducible Cre-mediated capability will be a powerful reagent for the investigation of cell proliferation in the context of *Aurkb* expression.

## Data Availability Statement

All datasets generated for this study are included in the article/Supplementary Material.

## Ethics Statement

The animal studies were reviewed and approved by the University of Pennsylvania Institutional Animal Care and Use Committee and the University of Maryland Baltimore Institutional Animal Care and Use Committee.

## Author Contributions

JE and DL conceived and designed the study. JJ, KE, and DL analyzed the experiments and wrote the original draft. JJ, KE, JE, and DL edited and finalized the manuscript. JJ, KE, FL, LL, GS, and DL performed the experiments. All authors reviewed the results and approved the final version of the manuscript.

## Conflict of Interest

The authors declare that the research was conducted in the absence of any commercial or financial relationships that could be construed as a potential conflict of interest.
