# Supplementary material for: An Engineered Mouse to Identify Proliferating Cells and Their Derivatives
Source: Front Cell Dev Biol. 2020 May 25;8:388. doi: 10.3389/fcell.2020.00388 (PMC7261916; doi:10.3389/fcell.2020.00388)
Supplement: Supplementary file 1 [file Data_Sheet_1.pdf]

Supplementary Material

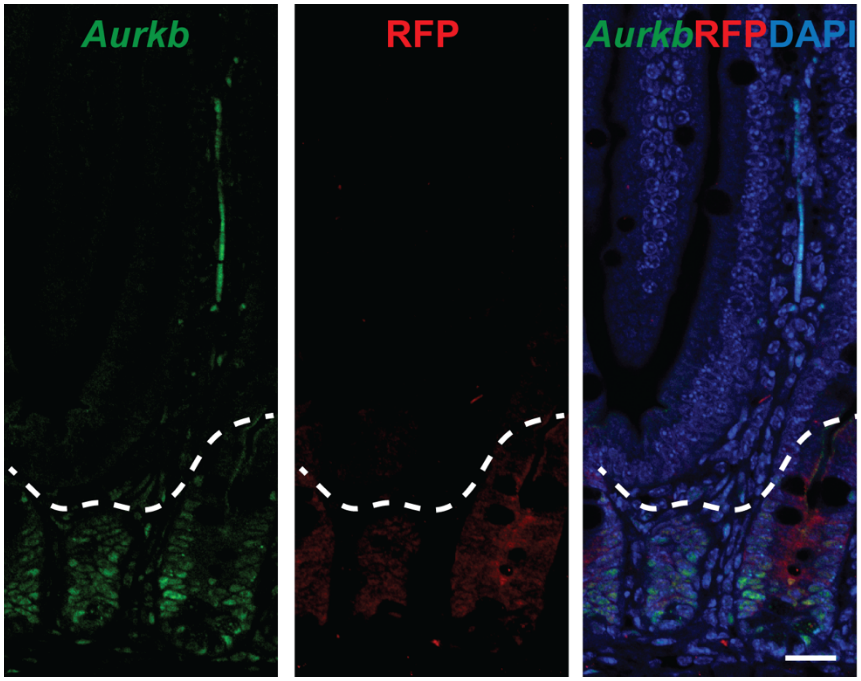

Supplementary Figure 1. Colocalization of Aurkb and tdTomato in adult intestinal crypts. Immunofluorescence staining of Aurkb and tdTomato was performed in 2-month-old *Aurkb*$^{ER\ Cre/+}$ intestine. The dotted lines delineate the intestinal cryptic zone. RFP antibody was used to recognized tdTomato signal. Scale bar, 25 μm.

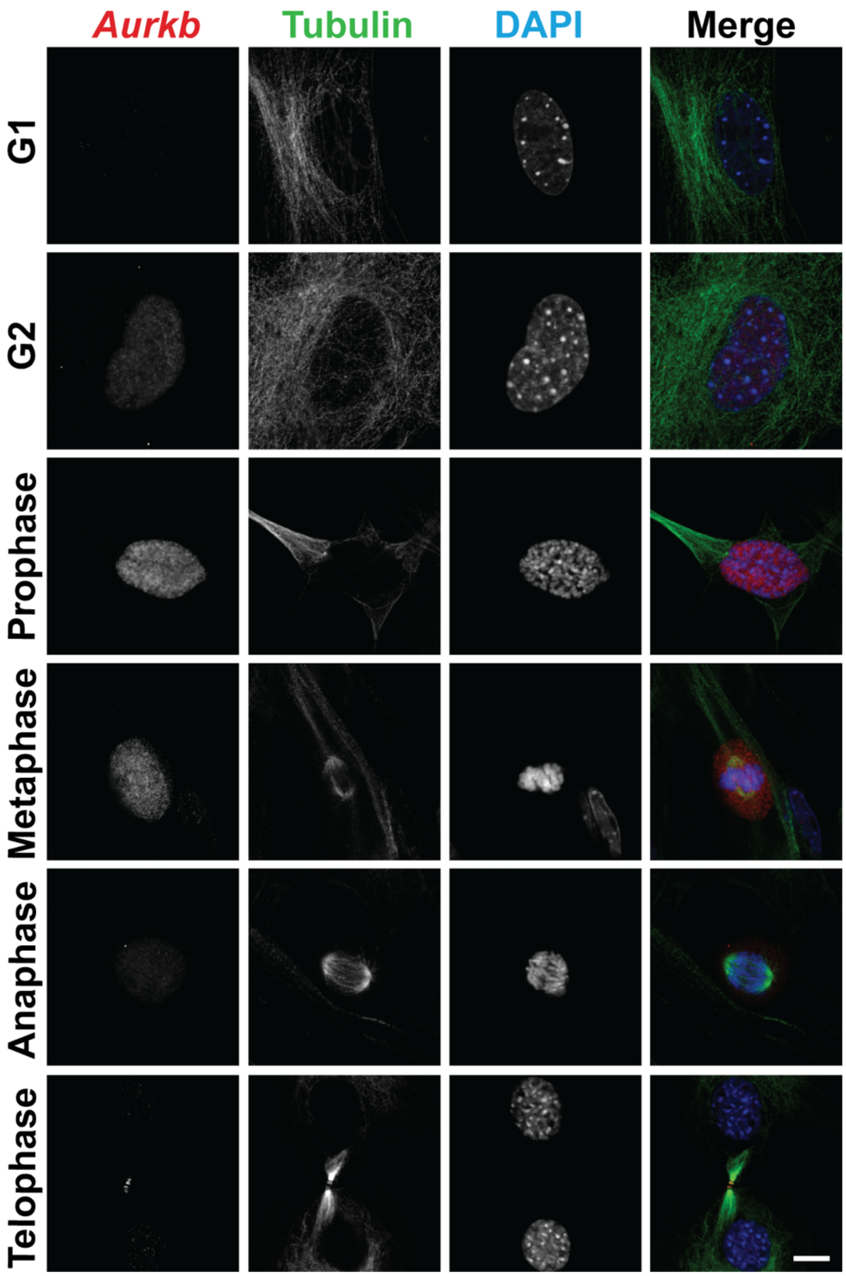

Supplementary Figure 2. Localization of Aurkb protein during the cell cycle. Immunofluorescence staining was performed in MEFs under normal culture conditions. Scale bar, 10 µm.

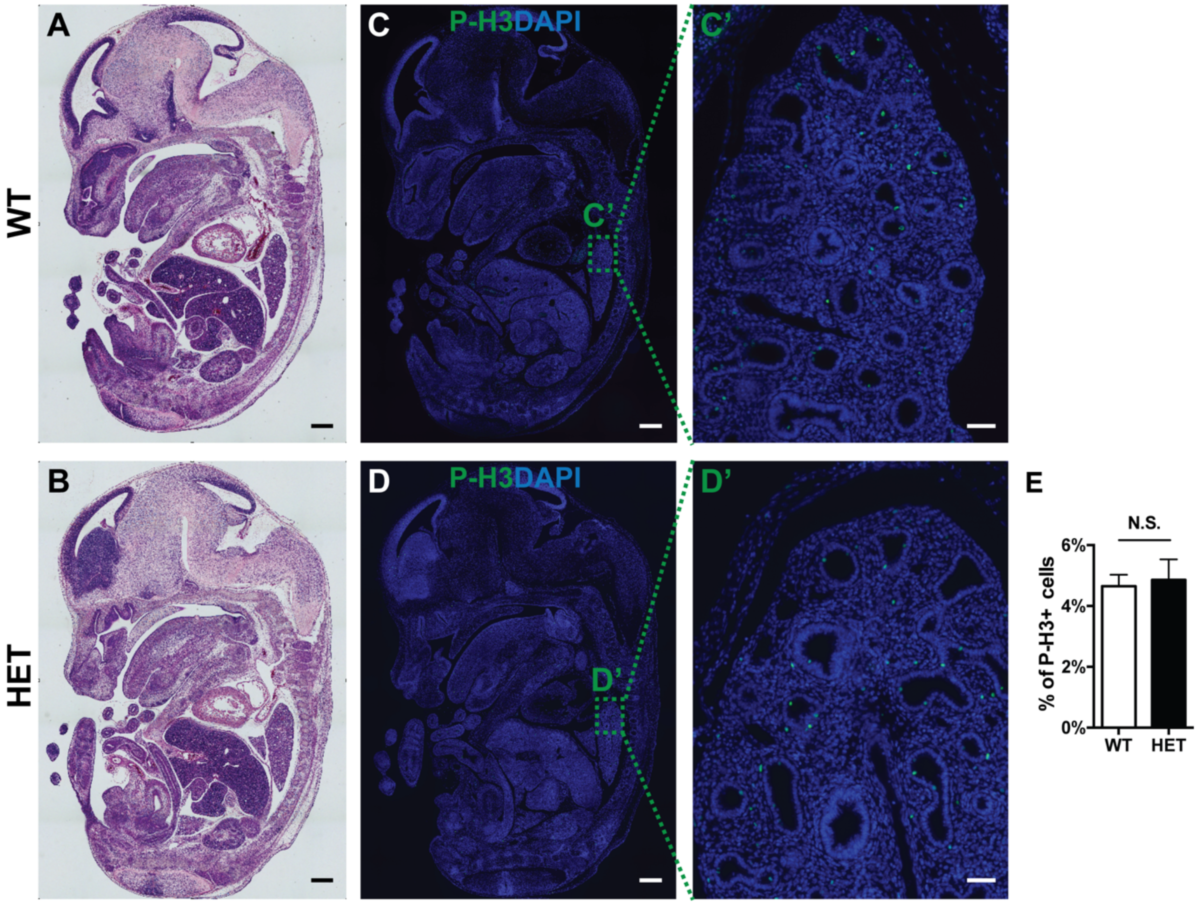

Supplementary Figure 3. Normal embryonic morphogenesis and growth in *Aurkb*$^{ER\ Cre/+}$ embryos. (A&B) Representative H&E staining micrographs of E14.5 wildtype (WT) and *Aurkb*$^{ER\ Cre/+}$ (HET) embryos; **(C&D)** Representative P-H3 immunofluorescence staining micrographs of E14.5 WT and HET embryos. Insets of C'&D' demonstrate P-H3 signals in embryonic lungs; **(E)** Quantification of the percentage of P-H3+ cells in WT (n=3) and HET (n=3) embryos. The percentage of P-H3+ cells was calculated by the total number of P-H3+ cells divided by the total number of embryonic cells (DAPI+). N.S., not statistically significant. Scale bars, A-D, 500 μm; C'&D', 50 μm.

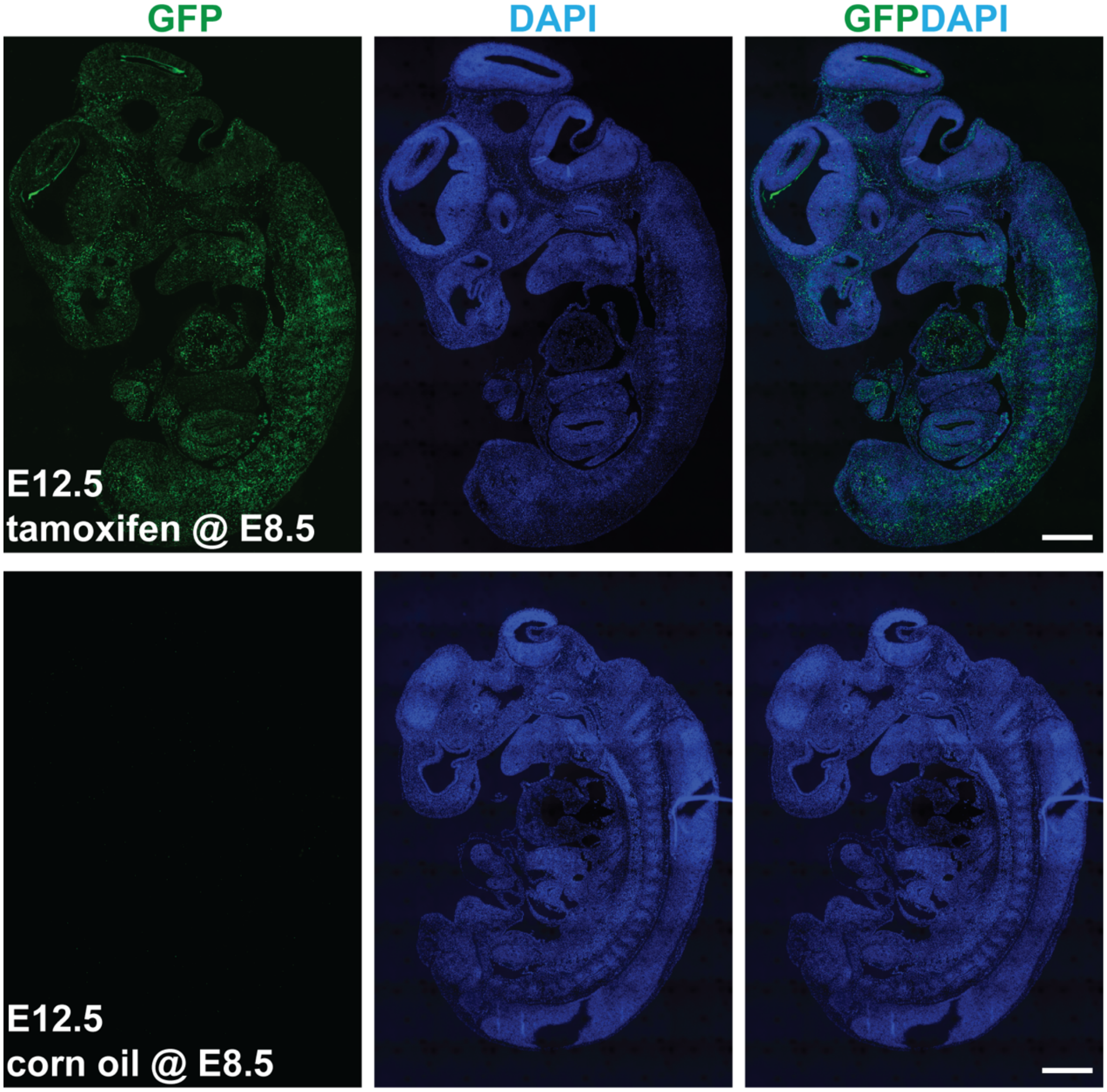

Supplementary Figure 4. Absence of leakiness of *Aurkb*$^{ER\ Cre/+}$ in developing embryos. Immunofluorescence staining of E12.5 *Aurkb*$^{ER\ Cre/+}$*; R26R*$^{eYFP/+}$ embryos (sagittal sections). Either tamoxifen (150 mg/kg BW, top row) or corn oil (150 µl/kg BW, bottom row) was given to pregnant mice at E8.5 by gavage. Scale bars, 500 µm.

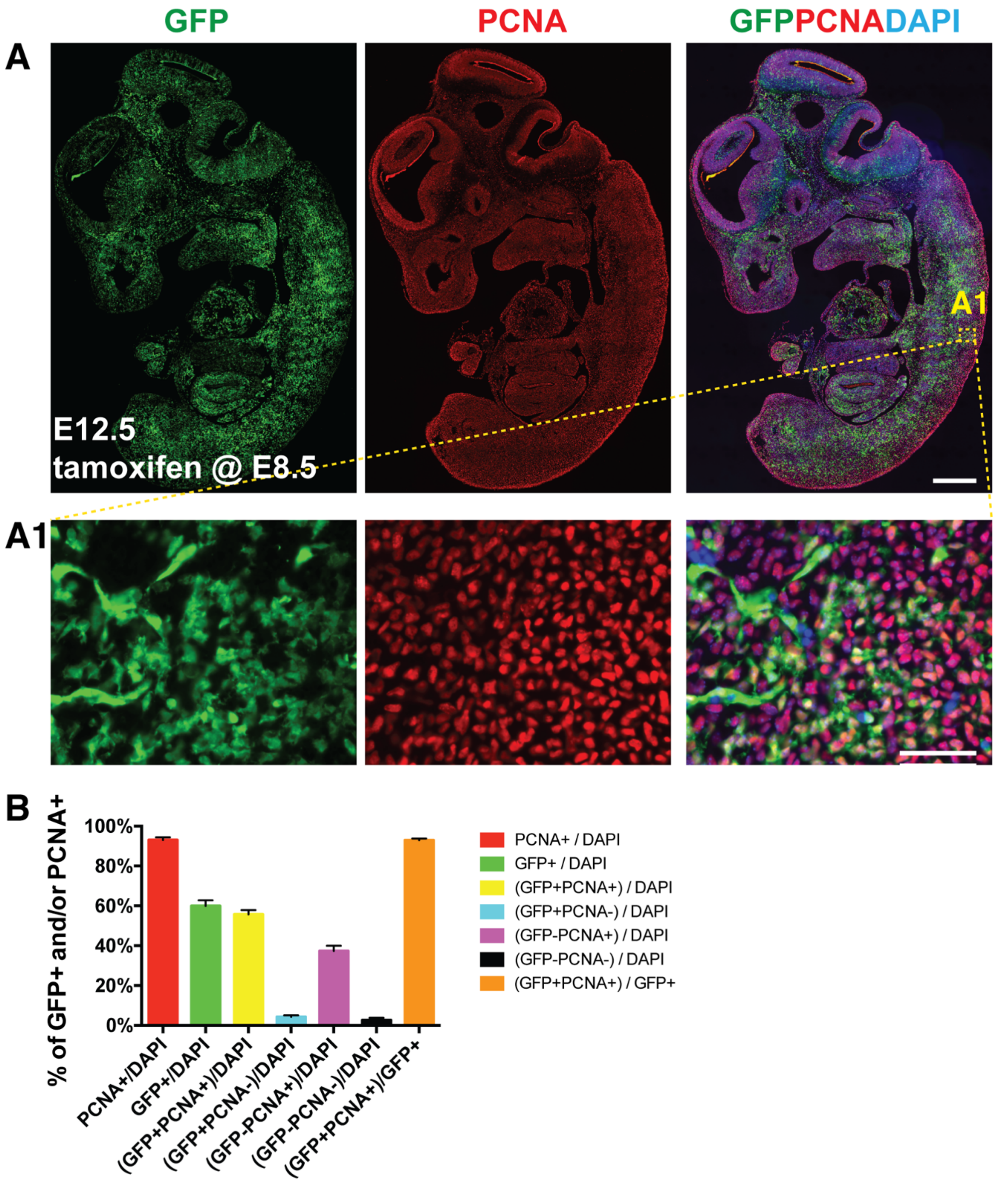

Supplementary Figure 5. Overlay of *Aurkb*$^{ER\ Cre/+}$ lineage tracing with PCNA in developing embryos. Tamoxifen (150 mg/kg BW) was given to pregnant mice at E8.5 by gavage. **(A)** Representative immunofluorescence image of an E12.5 *Aurkb*$^{ER\ Cre/+}$*; R26R*$^{eYFP/+}$ embryo (sagittal

section). Scale bars, A, 500 μm; A1, 50 μm; **(B)** Quantification of the percentage of GFP+ and/or the percentage of PCNA+ immunofluorescence staining (n=4).

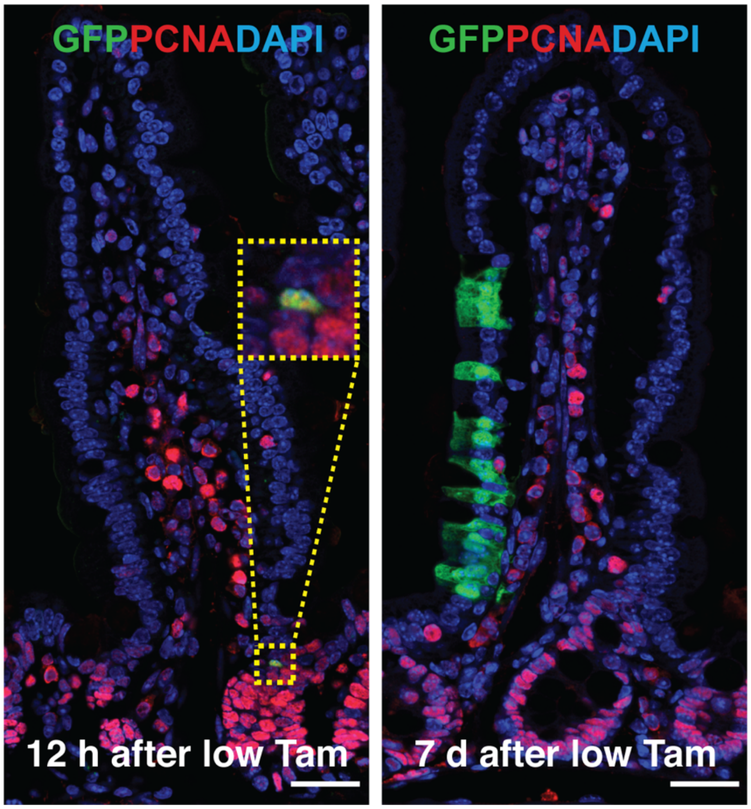

Supplementary Figure 6. Single earlier *Aurkb*-labeled intestinal progenitor cells give rise to a cluster of enterocytes. A single low dose of tamoxifen (2 mg/kg BW) was I.P. given to *Aurkb* $^{ER\ Cre/+}$*; R26R* $^{eYFP/+}$ mice at the age of 2 months. Tam, tamoxifen; h, hour; d, day. Bars, 25 um.

Supplementary Table 1. Antibodies used for Immunofluorescence

Antibody	Species	Catalog #	Vendor
GFP	Goat	AB6673	Abcam
GFP	Rabbit	2956s	Cell Signaling
RFP	Rabbit	600-401-379	Rockland
Aurkb	Rabbit	AB2255	Abcam
Cre	mouse	MAB3120	Millipore
β-tubulin	Rat	AB6160	Abcam
BrdU	Mouse	14-5071-80	Ebioscience
PCNA	Mouse	CM152B	Biocare
Ki67	Rabbit	AB16667	Abcam
P-H3	Mouse	9706S	Cell Signaling
MCM2	Rabbit	4007s	Cell Signaling
NeuN	Mouse	MAB377	Millipore

Supplementary Video 1. ***Aurkb*$^{ER\ Cre/+}$ labeling of a dividing MEFs.** *Aurkb*$^{ER\ Cre/+}$; *R26R*$^{eYFP/+}$ MEFs were cultured in 10% FBS standard medium inside of a 37°C IncuCyte live-cell culture system with humidified atmosphere of 5% CO_2. 4-OH tamoxifen (1 μM) was added to the medium, and time lapse phase-contrast and GFP (YFP signal can be detected by GFP channel) images were obtained over 24 hours. The expressed YFP signal was captured in the GFP channel. Sixteen hours after 4-OH tamoxifen treatment, GFP signal gradually turned on in the cell located in the middle of the field, and then it became intensified right before cytokinesis and stayed on in the two daughter cells. In contrast, two nondividing neighbor cells did not express GFP signal.
